# The Construction of Peer Support Among Recently Diagnosed Breast Cancer Patients

**DOI:** 10.1097/NCC.0000000000001319

**Published:** 2024-08-09

**Authors:** Anu Toija, Tarja Kettunen, Kirsti Kasila

**Affiliations:** Author Affiliations: Faculty of Sport and Health Sciences, University of Jyväskylä (Mrs Toija, and Drs Kettunen and Kasila), Finland; and Nursing Management, HUS Group (the Joint Authority for Helsinki and Uusimaa), Helsinki University Hospital and University of Helsinki (Mrs Toija), Finland.

**Keywords:** Breast cancer, Peer support, Self-determination, Thematic analysis

## Abstract

**Background:**

Breast cancer (BC) and its treatments decrease patients’ psychological well-being. Peer support is one form of social support, but little is known about what gives rise to peer support.

**Objective:**

The purpose of this study was to examine how peer support is constructed among recently diagnosed BC patients.

**Methods:**

Eighteen women were randomly picked from 130 women who had received phone calls from a trained peer supporter and were invited to group interviews. In the interviews, patients discussed their cancer, peer support experiences, and social support. The transcribed data were analyzed using Braun and Clarke’s thematic analysis approach.

**Results:**

The construction of peer support among newly diagnosed BC patients was complex. It depended on the needs of the patient and the success of interactions. Once they had received a diagnosis, the lives of the patients changed suddenly, and patients dove into the I-we-others consideration and had a need to talk. Interaction with peer supporters gave them a chance to share their stories. At their best, interactions led to belonging, caring, and a sense of security.

**Conclusions:**

The need to be heard and seen is strong in a patient’s changing health situation. Peer support plays an important role in high-standard care and in strengthening patients’ self-determination.

**Implications for Practice:**

Hospitals should create chances for supportive communication, and the supportive communication should be easily accessible and successful. The training of peer supporters should ensure that they have reflected on their own BC process and know how to consider the needs of newly diagnosed patients.

Breast cancer (BC) is the most common cancer among women all over the world.^1^ Breast cancer experiences and treatments have a wide range of effects on a patient’s physical, psychological, and social well-being and decrease the quality of life in every dimension.^2,3^ Although the survival rate is relatively good, the BC diagnosis challenges the central elements of well-being. Cancer and its treatments change the normal rhythm of daily life^4,5^ and agency in terms of ordinary activities and habits.^6^ A diagnosis of BC generates uncertainty about the future^4,7–9^ along with the fear of recurrence and death^4,9,10^ while changing social relationships.^4^ In addition, BC patients may feel stress and depression.^11^

Standard treatments of BC include surgery, radiation therapy, and chemotherapy, and these are planned and carried out with the individual in mind. Along with medical therapy, emotional and social supports are necessary components of high-standard cancer care.^12^ Breast cancer patients search for support from multiple social relationships, including family members and more distant relationships. More distant relationships influence stress, coping, and well-being.^13^ One form of social support is peer support, which refers to the support where individuals with a common lived experience share knowledge and emotional support reciprocally with each other. Peer support is based on sharing experiences and has many forms, from one-to-one interaction to group encounters, and it can be delivered, for example, face-to-face, via telephone, or online.^14,15^

Peer support can help patients learn more about their illness and compare and consolidate the information they have been given by professional caregivers.^16–21^ Peer support helps to put knowledge into practice and improves self-management skills^16^ and self-efficacy.^22^

Sharing personal narratives makes it possible to talk about one’s own experiences and to listen to survival stories,^17,23,24^ giving patients an understanding of what to expect and an opportunity to bond with positive role models.^23^ It helps patients to have an insight into other patients’ experiences, and it is also an important method to renegotiate patients’ own identity.^5^ Talking about one’s own experiences can be helpful for individuals in processing the meanings of experiences.^25^ When interacting with other BC patients and survivors, patients can get a feeling of normality and of belonging to something,^26^ and of being understood and accepted.^8,27^ Peer support also helps with emotional distress,^19,20,28^ and it has been noted to lead to less depression^29^ and fewer physical symptoms^19^ as well as better quality of life.^16,28,30,31^

However, we also know that interacting with peers does not always help.^31,32^ Reasons for the variable results of peer support have been found to be the level of moderation in the group discussions,^23,31^ the presence of^17^ and similarity with^33,34^ the peer supporter, and the level of participation activity.^20^ Yet, less is currently known about what gives rise to peer support and what kind of a role interaction and its dynamics play.

The previously mentioned literature review highlights how getting a BC diagnosis challenges one’s self-determination, which is a crucial element of well-being. Self-determination can be examined through psychological needs and how they are satisfied. According to self-determination theory, people’s innate psychological needs are autonomy, competence, and relatedness. Autonomy means being the source of one’s own behavior as well as being able to make choices and have control over one’s own life. Competence refers to a feeling of effectiveness or capability in ongoing interaction and experiencing opportunities. Relatedness, in turn, refers to a feeling of being connected to others as well as of caring for and being cared for by others. Psychological needs, both their frustration and satisfaction, are essential determinants of an individual’s well-being.^35–38^

In Finland, a major reformation of health and social services took effect at the beginning of 2023. The reorganization of services is still in progress, and the cooperation between hospitals and NGOs (nongovernmental organizations) is new. Although medical cancer care is provided by public services, peer support has mainly been organized by NGOs. In this study, the main objective was to combine the services of a hospital and NGOs so as to provide the most qualified care for BC patients. Peer support was offered for every patient as part of cancer care without the patients needing to be active in finding peer support. This is not a common protocol in Finland. As the results of previous studies concerning the effectiveness of peer support are contradictory, we need to determine what kind of interaction is crucial in making one-to-one peer support the most helpful. The purpose of this study was to delve into this issue and examine how peer support is constructed among recently diagnosed BC patients, especially from the interaction point of view.

## Methods

This study was part of a larger research project that carried out a simple and low-cost one-to-one peer support program for BC patients in cooperation between a Finnish hospital and NGOs.^33^ The study had approval from the HUS Helsinki University Hospital Ethical Committee (July 15, 2021 [HUS/1748/2021]), and the patients provided signed informed consent. Eighteen patients were randomly picked from 130 patients who had received 1 to 5 phone calls from a peer supporter and were invited to group interviews to discuss their peer support experiences in the new protocol. There were 5 different groups with 3 or 4 patients in each group.

During their first visit to the hospital, patients permitted their names and phone numbers to be given to the peer supporter, who then called the patients. The first phone call occurred at the time between diagnosis and the beginning of treatments. During the first phone call, peer supporters introduced themselves and explained why they were calling. Patients could choose what they wanted to discuss. Peer supporters were advised to make 1 to 5 phone calls, with about 1 week between the calls. Some of the patients wanted only 1 phone call, but some of them wanted to have as many as 9. At the end of each call, patients could choose whether they wanted to have another call. That is why some patients received the call only at the beginning of their treatments and some of them received the calls over a longer period. Peer supporters were the ones to make the call, but some peer supporters offered patients the opportunity to call as well.

The median age of the participants with BC was 62.5 years (range, 50–71 years). Interviews were carried out approximately 1 year after the end of their BC treatments. In this study, peer supporters (N = 15) were BC survivors trained in providing peer support. All peer supporters had had BC more than 2 years earlier. One-third of them were still working outside the home, and the others were retired. They all participated in the 16-hour peer support training program in the NGO in which they volunteered. The training program included processing one’s own experience of BC, the theory of peer support, interaction skills, and well-being as a peer supporter. In addition to that peer supporters gave patients 1 or more phone calls, the patients were encouraged to get in contact with cancer organizations.

In group interviews, patients freely discussed their BC and peer support experiences and social support in general. Interviews were carried out in Finnish, which was the patients’ mother tongue. Only the researcher and the patients were present in the interviews. Interviews were audio-recorded. The total duration of the 5 group interview sessions was 7 hours of audio material. They were transcribed verbatim into computer text files, 160 pages with 60 388 words. The patients were coded. For example, quote 1.1 means person 1 in group interview number 1. Quotes were translated into English and checked by 2 people, one of them a native English speaker.

The transcribed data were analyzed using Braun and Clarke’s^39^ thematic analysis approach. Thematic analysis is a data-driven inductive analysis that is used for exploring meaning patterns about the phenomenon under study. That is why it was chosen for this research.

Analysis was started by reading and rereading the transcripts to get familiar with the data. Meaningful units of peer support were then coded. There were no preset codes, but they were developed while working with the data. The codes discussing similar ideas or issues were next grouped into themes, with the themes highlighting important patterns across the data set concerning peer support. The analysis was conducted by the first author, and interpretations and their trustworthiness were reflected on together in regular meetings with all the authors. An example of the analysis process is illustrated in the Table. Through the analysis, the following themes were formed: I-we-others, the need to talk, interaction with peers, belonging, caring, and a sense of security.

**Table:** An Example of the Analysis Process

Meaning Unit/Quotation	Code	Theme
“No one who is not going down this path can truly know and cannot understand what is going on in your head!” (1.1)	Different from healthy people	I-we-others
“Everybody always has their own unique experience. We, too, have already heard how differently we are looking at these things.” (3.1)	Own unique experience in the group of BC patients	
“It’s really important that the someone you’re talking to is not a doctor, but someone you can call just like that.” (2.1)	Need to talk to someone other than a professional	Need to talk
“I don’t feel like burdening other people with my issues, and that’s why I talk it through with my peer” (2.1)	One doesn’t want to burden other people	

Abbreviation: BC, breast cancer.

## Results

**Figure 1:**
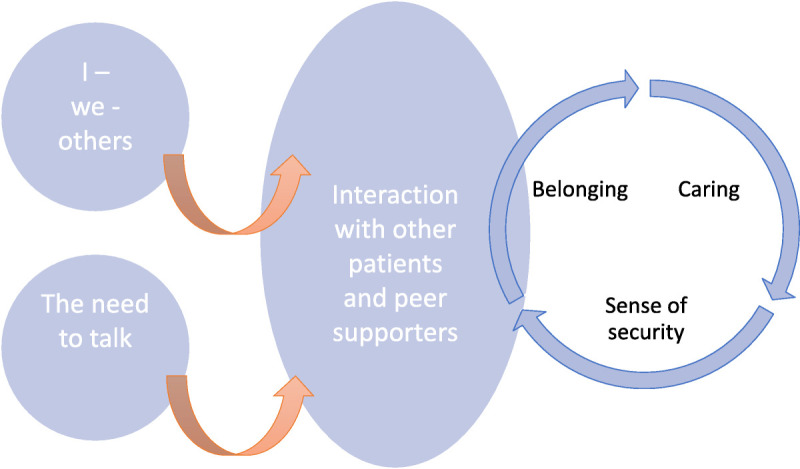
Critical elements in the construction of peer support.

The construction of peer support among newly diagnosed BC patients was complex. The BC experience felt unique. Peer support depended on the needs of the patient and the success of interaction. Once they had received a BC diagnosis, the lives of the patients changed suddenly. Interaction with other patients, survivors, and peer supporters gave them a chance to share their stories. Interaction was expected to be easily available and pleasant. The interaction between patients as well as between patients and peer supporters led to a sense of belonging; the patients felt cared for, and they had a sense of security, because they could deeply understand their own disease and how it could affect their life (Figure 1). When talking about peer support, patients referred to not only trained peer supporters but also other patients in the hospital or friends who had had BC.

## I-We-Others

For many patients, the BC diagnosis often came without warning. After receiving the diagnosis, patients had to wait for the treatments to begin. Patients described the waiting time as a long and fearful period, but on the other hand, it was also a time to gather information about BC and arrange their own affairs and thoughts. The diagnosis often led to not only shock, fear of death, and worries about one’s own condition but also concerns for loved ones such as children, a spouse, and parents. Sometimes, BC was only one thing to handle, and patients processed it by seeking information, continuing their lives, and working as normally as possible. After the treatments had started, time passed according to the timetables of the care. Sometimes treatments had many unwanted adverse effects, which affected the well-being. At times, patients’ physical and mental well-being varied.

Patients compared their experience in relation to other patients and survivors, and those who have not had BC. Patients felt that they were different from healthy people and not fully understood by them. This made newly diagnosed patients feel otherness from healthy people: “No one who is not going down this path can truly know and cannot understand what is going on in your head!” (1.1). Patients used the term “we” when referring to other BC patients and the term “others” when referring to healthy people. Patients had experiences that others, that is, healthy people, did not have, and they were searching for BC experiences of other patients and compared them with their own experiences. On the other hand, patients were aware of their own unique experiences in comparison with other BC patients and survivors as well. They emphasized their differences in perceiving situations, and one’s own experience was seen as a once-in-a-lifetime moment: “Everybody always has their own unique experience. We, too, have already heard how differently we are looking at these things” (3.1).

## The Need to Talk

The patients emphasized that it was self-evident that everyone who has BC should have someone to talk to—someone who listens and understands and can be easily contacted: “It’s really important that the someone you’re talking to is not a doctor, but someone you can call just like that” (2.1). Patients did not necessarily want to burden their loved ones with talking about their own worries: “I don’t feel like burdening other people with my issues, and that’s why I talk it through with my peer” (2.1). The need to talk was so self-evident that evaluating it was not considered appropriate: “And then she (peer supporter) asked at the end (of the phone call) that would I…want her to call me again. I was dumbstruck, like I wasn’t sure...you don’t really know what to say in a situation like that” (3.1). The patient seemed confused that she had to evaluate the need for peer support by herself, although it was part of the program that patients could make up their own minds about how many phone calls they would like to receive from a peer supporter.

## Interaction With Other Patients and Peer Supporters

When describing peer support, patients highlighted interaction with other patients whom they got familiar with during the hospital visits and with peer supporters. Patients wanted to reflect on one’s own experiences in many ways. They could read about BC experiences on the Internet and in books and interact with their acquaintances who had survived BC, other patients in the hospital, or peer supporters in the hospital and via telephone.

**Figure 2:**
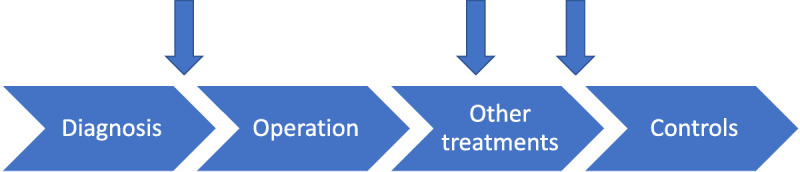
The moments when breast cancer patients felt the need for peer support in the timeline of care.

The ease of sharing stories was meaningful because these stories provided knowledge of patients’ diverse experiences. Sometimes getting knowledge from reading was enough, and patients felt they did not need to meet peer supporters. Patients also described moments during their care process when they longed for discussion with peer supporters. These moments were strongly related to daily life situations and emotions during the various periods of the care or illness process. Typically, these were described as relatively short moments (Figure 2). Peer support was needed during the times when patients required information and were feeling good or, on the other hand, when they were feeling unwell or lonely. These instances occurred especially immediately after receiving the diagnosis, during chemotherapy or radiotherapy, and after the end of treatments: “I missed (peer support) during chemotherapy. I would have needed it. Or at times when I was in really bad shape” (3.5). Although the patients may have felt they missed the peer support, they did not contact peer supporters on their own.

Interaction with other patients and peer supporters was the most convenient when they were part of everyday life. Sometimes finding peer support was effortless: if patients spoke openly about their experiences, they could find other BC patients or survivors. Sometimes it was easy for patients to get to know other patients in the hospital ward, to meet a peer supporter in a hospital, or to receive a phone call from the peer supporter, without taking the initiative. No effort had to be put into the encounters when they were part of the hospital visits: “Well, it all started at an appointment at the surgeon where the peer supporter was too. We started talking and it was easy for us to talk” (4.1).

When patients were with others who shared their experience, they felt understood, and they were allowed to talk about BC without restrictions, be tired, and express the spectrum of emotions. The patients perceived discussion with an unknown peer supporter as complementary to familiar relationships, because it was easy to talk to a stranger about difficult issues: “And that’s the first time since we started talking properly, she just asked if I had started planning my own funeral. We had good chemistry” (2.1). Shared experiences served to provide insight when patients could use dark humor. In the previous example, such humor seemed to strengthen the good chemistry between the peer supporter and the patient.

Although some patients longed to talk to other patients and peer supporters, they might not establish contact with others or might not receive a response even when doing so. It was not always easy to find other patients to talk to in the hospital. Patients’ personalities and their approaches influenced the ability to start a conversation with other patients. For those who described themselves to be shy, connecting with others was challenging: “I have to say that in the hospital where there were 10 of us…everyone was on their beds, and no one would talk to each other. Nobody…” (3.5).

Patients were familiar with the means to be in contact with peer supporters. Although the need to talk was great, contacting a peer supporter was a major threshold. Patients wondered why they had not taken the initiative to reach out to peer supporters, although they knew help was available. Seeking peer support felt laborious amid the fatigue, and sometimes patients downplayed their own worries. Sometimes they found it difficult to allow themselves to be in the position of being the supported one: “I was maybe pretending too much that I was OK and that there were no problems. That everything was fine. I was not receptive to help” (3.5).

In interaction with previously unknown peer supporters, building trust required time, and such trust that did build up was easily lost. Peer supporters faced great expectations. They were, for example, expected to have good interaction skills and to comply with the terms of the person being supported, be reliable, focus on listening, and not tell too much about their own life. The peer supporter was expected to be flexible and easy to reach. Trustworthiness was essential: “And well, then she said…she also has these difficult cases…she said she would call me in two weeks’ time and that two weeks never came” (1.5).

Although patients hoped the initiative would mainly come from the peer supporter, it was important that the peer supporter allowed the patients to play an active role, such as when agreeing on the schedules of the calls: “My peer supporter always made sure, even though we had agreed on calling every second or third week…on that same day or day before, she sent me an SMS and made sure it was OK that she called at so and so time” (1.4). However, scheduling seemed to be challenging. Calls sometimes came when it was inappropriate and not when they were needed.

There were also instances when the peer supporter failed to live up to expectations. The patients emphasized that a person with a recent diagnosis is in a delicate state and that the peer supporter must be very sensitive to how they receive that person. The beginning of the peer support discussions was the key. It was more difficult if the primary approach was made by phone when it was 2 previously unfamiliar persons talking for the first time: “Well, that’s probably what’s so difficult—that the person, chemistry…like how these two people who don’t know each other, how they eventually match” (2.4) and “The match then occurs based on discussions over longer periods of time” (1.1).

## Belonging, Caring, and a Sense of Security

A shared experience brought patients together as one. They felt a sense of belonging with the group of people with BC. Although the experience was not exactly the same, sharing stories felt meaningful. Such sharing made it possible to reflect on the experiences of others in light of their own and to find differences and similarities. The shared experiences with others reminded them of not being alone with their experience and tied them strongly together. They felt they had someone who fully understood their feelings and what they were going through: “Yes...I’m talkative and...I talk about my own things, and I like to hear...about the others’ situations...that’s really important...that’s why…it...kind of puts a perspective on it, that I’m not alone” (3.5).

When patients had been in contact with other patients, they often felt others cared for them. Other patients in the hospital were considered important, and the encounters with them were described with warmth. The other patients made the hospital visits significant: “Those chemotherapy treatments were such a lifeline for me, maybe because there were others in the same situation present, and then we talked about it quite freely” (1.1). Sometimes patients spontaneously formed a deeper peer relationship and kept in touch and cared for each other’s well-being: “and then if you don’t hear from the other for a while, you would immediately call and ask if they are OK” (3.1).

In this study, peer supporters called the patients on the phone. The time of the peer supporter’s call was remembered precisely. Patients were taken by the fact that peer supporters were interested in them. With a peer supporter, patients were able to be supported and play the main role in the interaction. Regular contacts and inquiries about well-being brought a sense of care. Even when the peer supporter contacted the patient according to the study protocol, it did not mean anything less. The fact that the contacts took place as part of the process brought a strong sense of care: “Well…someone cares about me [laughs]. And as a divorcee, I live alone…I was really pleased when strangers contacted me and were interested in me and me only [laughs]” (1.5).

At its best, the knowledge gained from peer interaction helped ease anxiety. The understanding shown by the other patients and peer supporters and the convincing knowledge from lived experiences, as well as the increased understanding of patients’ own situation and individual variations, reduced fears: “Well, I feel I benefitted, that I didn’t get scared straight away, because I asked her straight away if she had this or that symptom, and she told me whether she had it or not, and realizing it was different for all of us—that there was variation. Knowing that has calmed me” (3.1).

The presence of a peer supporter in the hospital brought peace to that moment. Seeing a peer supporter made it clear that the future can look alive and active and that one can survive BC. Discussion and understanding calmed patients’ minds:

(1.2) “From my experience, when I went to the surgeon for the first time for the breast treatment section, she had this reassuring presence about her, bringing peace to the situation.”

(Interviewer) “So, the peer supporter was there?”

(1.2) “Yes, she was there. Yes, and then when everything was like…well it was….”

(4.2) “There was a kind of peculiar vibe in there.”

(1.2) “Yes, that’s it. There were us women sitting there, and….”

(4.2) “There was a queue already.”

(1.2) “This peer supporter was there, chatting casually.”

(4.2) “Yes.”

(1.2) “Just like we are here.”

(4.2) “Exactly.”

(1.2) “Yes, I find it calming that way. I had really positive thoughts about it, about being in the ward and having surgery and everything.”

Identification with the peer supporters was important in maintaining the hope of survival. Interacting with peer supporters brought hope for the future when patients saw the other person had survived an even worse situation than they were at the time. With the help of peer supporters, patients learned to prepare for what the disease might bring. Even unpleasant experiences served as preparation for the future.

(1.5) “It’s probably just that you can believe them in a completely different way...And the other one says...Whoever has gone through….”

(2.5) “Has been through it.”

(1.5) “Through.”

(2.5) “Through yes.”

(1.5) “Makes one feel like…More calm…Feeling like…it’s like…Also, when thinking of the future.”

## Discussion

This study described the construction of peer support among recently diagnosed BC patients, especially from the interaction point of view. Peer support was constructed by the patients’ needs, interaction with other patients and peer supporters, and the satisfaction of those needs. The BC diagnosis led to a sudden unpredictable life change that challenged the fulfillment of patients’ psychological needs. Patients had a need to reflect on their identity and a need to talk. These needs were changing at times and depended on patients’ well-being. Successful interaction with other patients and peer supporters played a central role in making peer support possible. Patients interacted with other BC patients, survivors, and peer supporters in multiple ways, and according to the results, interaction could contribute to belonging, caring, and a sense of security.

The data of this study were analyzed using inductive thematic analysis. In this section, the significance of these results is brought out by reflecting on them through 3 basic psychological needs—autonomy, competence, and relatedness—which are based on self-determination theory.^34,35^ When falling ill with BC, an individual’s power and capability to have control over their life deteriorates. However, successful peer support can strengthen the sense of self-determination.

According to this study, successful interaction with other patients and peer supporters was essential in the construction of peer support. Surprisingly, patients were thankful for peer supporters yet critical of the quality of their communication. The expectations for peer supporters were high. Interaction with peer supporters was vulnerable and prone to failure if the quality of interaction did not meet the expectations. The willingness to control communication as much as possible in interaction was highlighted. However, the willingness and capability of having control were changing. Autonomy in the interaction was enabled through small actions. Patients’ autonomy was supported by the possibility to choose when and how to be contacted, and the dialogue should ideally be built on the terms of the newly diagnosed person. Successful interaction was formed through negotiation between the patient and the peer supporter during the communication.

Our results suggest that getting a BC diagnosis inevitably affects patients’ experience of their autonomy. Holmberg^4^ found similar results: patients are no longer in charge of the pace of everyday activities, as BC and its treatments take a lot of time from daily life. In cancer care, the timetable is tight and inflexible, an aspect that may have contributed to the importance of patients making their own decisions regarding the timing and content of peer relationships. As a result of this study, negotiation was key to successful interaction, a finding that resonates with self-determination theory,^37^ where it is stated that being autonomous does not mean being independent but being supported. In this study, the degree of support was about moving sensitively in a fragile space between giving enough support and giving enough space for self-determination. This sensitivity was also highlighted in Holmberg’s^6^ study, where the agency was changing from stable to fragile or recreated agency. Breast cancer care challenges the autonomy of patients, meaning it was even more important for peer supporters to act sensitively and in an autonomy-supportive way in their interaction with patients. Peer supporters must focus on listening to patients and acting according to their wishes. To make this possible, it is important that peer supporters have reflected on their own experiences of BC as well as that they possess good communication skills. These needs further highlight the importance of peer support training.

This study revealed that peer support is also constructed by a sense of security. Interacting with other patients and peer supporters helped to create a sense of security in the middle of a frightening illness that made the future uncertain. Patients expected to be fully understood and find hope for their situation. To gain information, knowledge of the disease and its treatments was necessary. However, peer support was not only about information but also about understanding what was happening on an emotional level as well as about gaining hope and preparing for the future. To do this, patients used multiple ways to share stories with other patients and peer supporters. The means to enable sharing stories were multifaceted, and in addition, the amount of interaction varied at times. Talking about an altered and unfamiliar life situation was especially meaningful, and listening to the experiences of others was found to be helpful. Patients described that it was a relief to hear about a range of BC experiences. There was a slight contradiction in that patients showed a need to hear and read about others’ experiences, but peer supporters were not expected to say too much about their personal experiences. Moreover, although family members were often supportive and listened, patients did not want to burden their loved ones and they were relieved to be able to talk freely to peer supporters.

When we compare the results with other studies, we see similar results. For example, peer supporters’ knowledge provides extra value to the information given by doctors and nurses. According to studies of BC and peer support,^16–21^ patients get more understandable information and can consolidate it better with the help of peer supporters. Other studies have also shown that it is not necessary to find another person with exactly the same experience.^5^ Instead, what is important is for patients to hear about different experiences and survival stories as well as to reflect on their own experiences.^17,23,24,33^ In addition, Vygotsky’s^25^ theory of inner speech suggests that when individuals talk aloud about their own experiences, it is easier to understand them. It is important to note that understanding BC gives patients a more secure feeling. Similar to Holmberg,^10^ we found that interacting with peer supporters helped to create a sense of security in the middle of a frightening illness that made the future uncertain. This suggests that when patients feel they understand themselves and their illness, it gives them a sense of security. This sense of security, in turn, resonates with an important psychological need called competence,^38^ and peer support is one essential method of meeting that need.

According to this study, the experience of differentiating from healthy people and belonging to BC patients was important in the construction of peer support. Breast cancer diagnosis led to the need of renegotiating one’s own identity, from where to differentiate and where to belong. Patients reflected on their own identity in 3 ways: compared with healthy people and compared with other patients and peer supporters. Feeling different from healthy people and having a special experience compared with healthy people made patients more sensitive to becoming understood and feeling normal with other patients. These comparisons and the identity work the patients made reinforced the feeling of belonging to other patients. Patients also felt they were cared for by other patients and peer supporters. Patients were delighted when peer supporters contacted them and asked how they were doing and when other patients seemed to enjoy their company.

Similar to differentiation, in other BC studies, patients have been recognized as dealing with feelings of isolation^12^ and changes in social relationships.^4,6^ Similar findings have been reported in Mohlin and Bernhardsson’s^5^ study of the narratives of BC survivorship, where they observed that patients struggle between feeling of being alone and, at the same time, being part of a wider network. In addition to using the concept of belonging,^26^ peer support has also been described as helping feel normality and being accepted.^8,26,27^ Differentiation can be seen as the frustration of the psychological need for relatedness. According to self-determination theory, relatedness is satisfied by connecting with and feeling significant to others.^35,38^ In this study, the patients felt both belonging and caring with peers, and through this, an unsatisfied need for relatedness began to be fulfilled.

On the basis of this research, it would seem that the essential factors in constructing peer support among BC patients are successful interaction between BC patients and peer supporters, differentiation from healthy people and feeling a sense of belonging and caring with other people with experience of BC, and the sense of security that was gained by interaction with peers. These elements are crucial in satisfying the innate psychological needs of autonomy, competence, and relatedness, which in turn strengthen the sense of self-determination.

## Strengths and Limitations

This study had both strengths and limitations. Strengths include the number of participants, which was sufficient for qualitative research, and how the group interviews produced a rich variety of material. The material also contained many emotional-level descriptions. When examining the results, it is important to consider the phenomenon’s cultural and gender connections, because they may affect patients’ attitudes toward being supported. Different kinds of diseases other than cancer can also affect the way self-determination is threatened. Future research on the topic of peer support should be carried out in other cultures, on other diseases, and with different genders. More research is needed on what is successful interaction between peer supporters and patients, especially listening skills and how the potential for successful interaction can be improved.

## Conclusions

This study gives important tools in developing a new protocol for combining the services of a hospital and NGOs to provide the most qualified care for BC patients. The results of this study increase understanding of how peer support is constructed among newly diagnosed BC patients and what expectations are placed on peer supporters. It also suggests that peer supporters should be trained in how to meet patients’ expectations. Moreover, the study shows that peer support plays an important role in strengthening patients’ self-determination. The sense of competence may be supported by strengthening the feeling of security that is gained from knowledge and understanding of the disease and by identification with survivors. The sense of relatedness may be strengthened by increasing the feeling of belonging to the group of BC patients and by the caring that is expressed among those who have a shared experience. In addition, the feeling of autonomy can be supported via successful interaction with peer supporters, where patients’ agency is maximized and enabled within the limits of their current state of health and functional capacity. Peer support plays a critical role in high-standard BC care. The need to be heard and seen is strong in a BC patient’s changing health situation, a need that should automatically be considered in the healthcare system.

There is a need to increase the general understanding of the importance of interaction among those who share similar experiences. Hospitals should develop new practices to create possibilities for patients to discuss with each other and peer supporters. This new way to help patients, where professionals and lay workers cooperate more closely, requires trust. In the cooperation between hospitals and NGOs, the nurses’ role is to build trust in the help of nonprofessionals by making peer support interaction possible in hospital settings and encouraging patients to get involved with peer support activities. Overall, more people should be given the opportunity to experience peer support as well as to access alternative methods to obtain such support. Supportive communication should be easily accessible to all. In the future, more attention should be paid to the training of peer supporters to ensure they have reflected on their own BC process and know how to consider the needs of newly diagnosed patients as well as that they possess the ability to listen to others.
